# Tumortherapieassoziierte neurologische Symptome

**DOI:** 10.1007/s00115-021-01223-9

**Published:** 2021-11-25

**Authors:** Mirjam Renovanz, Johannes Rieger, Ghazaleh Tabatabai

**Affiliations:** 1 Abteilung Neurologie mit interdisziplinärem Schwerpunkt Neuroonkologie, Universitätsklinikum Tübingen, Hertie-Institut für Klinische Hirnforschung, Eberhard-Karls-Universität Tübingen, Tübingen, Deutschland; 2 Zentrum für Neuroonkologie, Comprehensive Cancer Center Tübingen-Stuttgart, Universitätsklinikum Tübingen, Eberhard-Karls-Universität Tübingen, Tübingen, Deutschland

**Keywords:** Neurotoxizität, Zentralnervensystem, Peripheres Nervensystem, Enzephalopathie, Myopathie, Neurotoxicity, Central nervous system, Peripheral nervous system, Encephalopathy, Myopathy

## Abstract

Die onkologische Behandlung ist biomarkerbasierter, molekular maßgeschneiderter und effektiver geworden. Aufbauend auf der zunehmenden Entschlüsselung zellbiologischer und molekularer Mechanismen steigt auch die Zahl zielgerichteter medikamentöser Therapien. Es steigt zudem die Zahl der Langzeitüberlebenden. Eine neuro(onko)logische Betreuung wird immer wichtiger, nicht nur wegen vermehrter direkter tumorbedingter Symptome – wie etwa der höheren Inzidenz einer Metastasierung in das Zentralnervensystem –, sondern weil im Zuge dieser modernen onkologischen systemischen Therapieformen ein breites Spektrum therapieassoziierter neurologischer Symptome auftritt, die einer sorgfältigen und raschen neurologischen/neuroonkologischen Evaluation und Therapiekonzeption bedürfen. Das Ziel dieses Artikels ist es, das Bewusstsein für die häufigsten therapieassoziierten neurologischen Symptome zu schärfen.

## Abstract (en)

Systemic treatment in oncology has become more biomarker-based, molecularly tailored and effective. Building on increasing scientific insights into cell biological and molecular mechanisms, the number of targeted drug therapies is also increasing. There is also an increase in the number of long-term survivors. Neuro(onco)logical care is becoming increasingly more important, not only because of increased direct tumor-related symptoms, such as higher incidence of central nervous system metastases, but also because a broad spectrum of treatment-associated neurological symptoms occur during the course of these modern oncological systemic therapies, which require careful and fast neurological/neuro-oncological evaluation and treatment. The goal of this article is to raise awareness of the most common treatment-associated neurologic symptoms.

## Lernziele

Nach der Lektüre dieses Beitrages …

- kennen Sie die tumortherapieassoziierten neurologische Symptome des zentralen und peripheren Nervensystems,
- wissen Sie, wie je nach Schwere der Symptome systematisch klinisch und diagnostisch vorzugehen ist,
- sind Sie über das Vorgehen bei selteneren therapieassoziierten neurologischen Syndromen informiert,
- wissen Sie, welche Therapiestrategien anzuwenden sind.

## Hintergrund

Die therapieassoziierten neurologischen Symptome moderner onkologischer Therapien gehen über die langjährig bekannten chemotherapieassoziierten Polyneuropathien hinaus. Das liegt daran, dass zunehmend **zielgerichtete Therapiestrategien**

zielgerichtete Therapiestrategien

angewendet werden. Sie greifen spezifisch an speziellen Molekülen oder **Signaltransduktionswegen**

Signaltransduktionswegen

an, deren Bedeutung für die Krebsentstehung und -fortdauer durch grundlagenwissenschaftliche Arbeiten im Laufe der letzten Jahre aufgeklärt wurde. Viele dieser **molekularen Zielstrukturen**

molekularen Zielstrukturen

liegen auch in Kompartimenten des zentralen und/oder peripheren Nervensystems und der Muskulatur vor. Eine Modifikation dieser Moleküle und Signaltransduktionswege im Rahmen zielgerichteter Tumortherapien behandeln folglich nicht nur die Tumorerkrankung, sondern führen auch zu therapieassoziierten neurologischen Symptomen.

Zuletzt stark in den klinischen Fokus gerückt sind neurologische Komplikationen bei **Checkpoint-Inhibitoren**

Checkpoint-Inhibitoren,

die z. B. in der Behandlung des malignen Melanoms eingesetzt werden. Therapieassoziierte neurologische Symptome treten mit einer Inzidenz von 3,8 % bei **Anti-CTLA4**

Anti-CTLA4

(„cytotoxic T lymphocyte-associated antigen 4“)-Therapie (z. B. Ipilimumab), 6,1 % bei **Anti-PD‑1**

Anti-PD‑1

(„programmed cell death protein 1“)Antikörpern (z. B. Nivolumab und Pembrolizumab) bzw. 12 % bei einer Kombinationstherapie auf [1, 2]. Weitere Therapien, die im klinischen Alltag häufig verwendet werden und zu neurologischen Symptomen bzw. deren Aggravation führen können, sind z. B. Temozolomid, Procarbazin, Vincristin, Lomustin, Bevacizumab, Marizomib und „Chimeric-antigen-receptor“(CAR)-T-Zellen [3].

## Systematisches klinisches und diagnostisches Vorgehen

**Figure 1:**
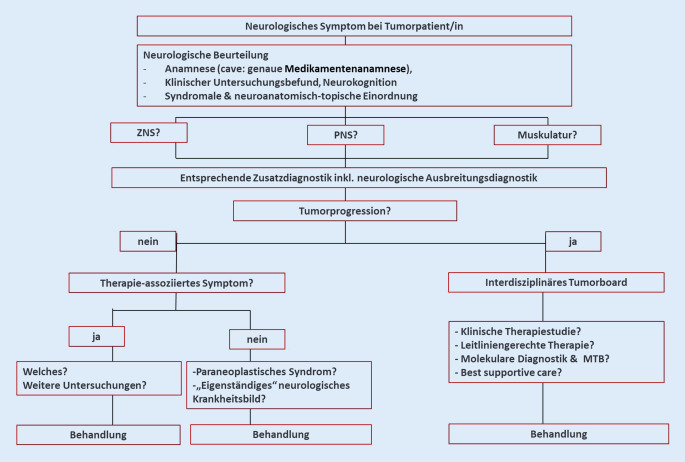

Für den klinischen Alltag bedeutet dies, dass Neurologinnen und Neurologen zunehmend mit neurologischen Symptomen onkologischer Patientinnen und Patienten konfrontiert werden und um Mitbeurteilung und Mitbehandlung gebeten werden [1, 4]. Bei **neurologischer Vorstellung**

neurologischer Vorstellung

eines Tumorpatienten/einer Tumorpatientin muss eine zügige und systematische Abklärung erfolgen, um zwischen tumorbedingter und therapieassoziierter oder auch anderer Ursache unterscheiden zu können (Abb. 1).

Die **neurologische Anamneseerhebung**

neurologische Anamneseerhebung

und klinische Untersuchung erfolgt wie üblich, eine sehr sorgfältige **Medikamentenanamnese**

Medikamentenanamnese

ist für die **differenzialdiagnostische Abklärung**

differenzialdiagnostische Abklärung

besonders essenziell, und basierend auf der neuroanatomisch-topischen Zuordnung kann die laborchemische, Liquor- und apparative Zusatzdiagnostik in die Wege geleitet werden.

Eine wichtige initial zu klärende Frage ist, ob die neurologische Symptomatik tumorbedingt ist, also Ausdruck einer Tumorprogression ist. Tumortherapieassoziierte Symptome treten üblicherweise 3 bis 12 Wochen nach dem Beginn der Therapie auf, selten sogar später [5, 6]. Daher sind eine sorgfältige **neurologische Ausbreitungsdiagnostik**

neurologische Ausbreitungsdiagnostik

inklusive zerebraler und spinaler Magnetresonanztomographie (MRT) und Liquordiagnostik wichtige diagnostische Maßnahmen.

Liegt eine Tumorprogression vor, sollten die weiteren Schritte im **interdisziplinären Tumorboard**

interdisziplinären Tumorboard

diskutiert werden. Insbesondere ist dabei natürlich zu prüfen, ob eine Behandlung im Rahmen klinischer Studien oder leitliniengerecht verfügbar ist. Sollte dies nicht möglich sein, kann in spezialisierten Zentren eine erweiterte molekulare Diagnostik erfolgen, um durch das molekulare Profil mögliche zielgerichtete Therapiestrategien auswählen zu können. Diese können dann im **„molekularen Tumorboard“**

„molekularen Tumorboard“

(MTB) priorisiert und anschließend im interdisziplinären Tumorboard im Hinblick auf die Einsetzbarkeit diskutiert werden. Je nach klinischem Zustand sind ggf. auch eine Deeskalation der Tumortherapie und eine symptomorientierte Behandlung indiziert.

Falls keine direkte tumorbedingte Ursache der neurologischen Symptome eruierbar ist, sind therapieassoziierte neurologische Symptome, ein paraneoplastisches neurologisches Syndrom oder eine **andere „eigenständige“ neurologische Zweiterkrankung**

andere „eigenständige“ neurologische Zweiterkrankung

in Erwägung zu ziehen.

Im Folgenden liegt der Fokus dieses Artikels auf den häufigsten therapieassoziierten neurologischen Symptomen. Wir weisen darauf hin, dass sich Behandlungsstrategien in diesem Bereich fortlaufend ändern können, sodass wir empfehlen, bei entsprechender klinischer Arbeitshypothese sehr zügig die diagnostischen Wege einzuleiten – je nach Dynamik und Schwere der Symptomatik im Rahmen einer stationären Einweisung – und die Behandlung gemeinsam mit einem **zertifizierten neuroonkologischen Zentrum**

zertifizierten neuroonkologischen Zentrum

durchzuführen.

### Das Wichtigste für immuntherapieassoziierte Nebenwirkungen auf einen Blick

- Therapieassoziierte neurologische Symptome treten meist zwischen 3 und 12 Wochen nach Beginn der Therapie auf [5, 6], individuell kann dies jedoch deutlich abweichen.
- Die Einteilung erfolgt nach Common Terminology Criteria for Adverse Events (CTC AE).
- Bei der differenzialdiagnostischen Abklärung sind vor allem Tumorprogression, epileptische Anfälle, Infektionen und metabolische Ursachen in Betracht zu ziehen.
- Bei milden neurologischen Symptomen (CTC-AE-Grad I, mild) sollte die Immuntherapie pausiert werden, bis alle weiteren möglichen Ursachen abgeklärt wurden.
- Prednisolon 0,5–1 mg/kg Körpergewicht (KG) oral wird für die Behandlung moderater Symptome empfohlen (CTC-AE-Grad II).
- Bei mittelschweren Symptomen (CTC-AE-Grad III) sollte Prednisolon 1–2 mg/kgKG i.v. verabreicht werden, bei schwer (CTC-AE-Grad IV) betroffenen PatientInnen kann auch eine höher dosierte Behandlung mit Methylprednisolon mit 500–1000 mg über 3 bis 5 Tage gewählt werden.
- Intravenöse Immunglobuline (IVIG) und Plasmapherese sind vor allem bei myasthenem Syndrom, Guillain-Barré-Syndrom (GBS) und transverser Myelitis indiziert.
- Die ZNS(Zentralnervensystem)-Toxizität nach Behandlung mit CAR-T-Zellen muss sehr genau diagnostisch eingeordnet werden, ggf. ist eine Therapie mit Tocilizumab, einem gegen den Interleukin-6-Rezeptor gerichteten Antikörper, einzusetzen [3].

#### Merke

- Es kommt auf eine zügige Diagnostik und rasche Einleitung der Therapie an.
- In der Regel gilt für die Behandlung immuntherapieassoziierter neurologischer Nebenwirkungen „steroide first“.
- Eine sehr enge und hochfrequente interdisziplinäre Abstimmung ist entscheidend (z. B. mit der primär behandelnden onkologischen Disziplin sowie ggf. weiteren Disziplinen bei Multiorganbeteiligung).

## Peripheres Nervensystem und Muskulatur

Das periphere Nervensystem und die Muskulatur sind gerade bei den immuntherapieassoziierten neurologischen Nebenwirkungen häufiger betroffen als das zentrale Nervensystem.

### (Poly‑)Neuro- und Radikulopathie

Eine Vielzahl zielgerichteter Therapien kann zu **peripheren Neuropathien**

peripheren Neuropathien

führen. Für die **klassischen Zytostatika**

klassischen Zytostatika

wie Vincristin oder Taxane oder Platinderivate ist dies schon lange bekannt, unter den modernen Therapien gilt dies z. B. für **Proteasominhibitoren**

Proteasominhibitoren,

**PD-1-Antikörper**

PD-1-Antikörper

und PD-L1(„programmed death-ligand 1“)-Antikörper (z. B. Durvalumab, Atezolizumab und Avelumab), weitere immunmodulatorische Substanzen sowie Antikörper-Wirkstoff-Konjugate [7]. Die Symptome bleiben oft auch nach Abschluss der Behandlung bestehen und beeinträchtigen die langfristige Funktion und Lebensqualität [7].

Für den klinischen Alltag ist zu beachten, dass eine vorbestehende **diabetische Neuropathie**

diabetische Neuropathie

natürlich die Effekte nicht nur maskieren, sondern auch verstärken kann. Es scheint so zu sein, dass tumorassoziierte Neuropathien bei diabetischen PatientInnen länger anhalten. In einer rezenten Studie persistierte die periphere Neuropathie bei einem höheren Anteil der diabetischen PatientInnen (im Vergleich zu nichtdiabetischen PatientInnen) bis zu 2 Jahre nach der Behandlung. Es wird sicher wichtig sein, in künftigen Studien das Zusammenspiel diabetischer Neuropathien und tumorassoziierter Neuropathien weiter herauszuarbeiten. Wichtige Parameter könnten hierbei z. B. die Form und Dauer des Diabetes sowie die **antidiabetische Therapie**

antidiabetische Therapie

sein [8].

Bei der tumortherapieassoziierten Polyneuropathie sind meist **Dosisreduktion**

Dosisreduktion,

Therapiepause bis hin zu **Therapieabbruch**

Therapieabbruch

die einzige Strategie zur Verhinderung schwerer tumortherapieassoziierter Neuropathien, was im klinischen Alltag oft herausfordernd ist: Wie soll ein Gleichgewicht gefunden werden zwischen maximaler Behandlungsexposition und minimalen langfristigen Nebenwirkungen? Es wäre daher empfehlenswert, bereits vor dem Start der onkologischen Therapie eine neurologische und **neurographische Untersuchung**

neurographische Untersuchung

als Ausgangsbefund durchzuführen und diese u. U. auch im Verlauf regelmäßig zu wiederholen. Ein **neurologisches Monitoring**

neurologisches Monitoring

würde es sicher leichter ermöglichen, die Behandlung durch eine engmaschigere Überwachung von RisikopatientInnen zu personalisieren [9]. Hierzu ist eine enge **interdisziplinäre Zusammenarbeit**

interdisziplinäre Zusammenarbeit

erforderlich. An dieser Stelle sei der Vollständigkeit halber darauf hingewiesen, dass es noch **keine evidenzbasierte Prophylaxe**

keine evidenzbasierte Prophylaxe

für eine tumortherapieassoziierte Neuropathie gibt. Die bisherige Literatur zu **Vitamin E**

Vitamin E,

das gehäuft eingesetzt wurde, zeigt keine überzeugenden Belege hierfür: Ergebnisse doppelblinder randomisierter klinischer Studien deuten jedenfalls darauf hin, dass Vitamin E die Inzidenz tumortherapieassoziierter Neuropathien nicht signifikant reduzierte. Zudem gab es auch keinen signifikanten Unterschied in der Häufigkeit schwerer tumortherapieassoziierter Neuropathien zwischen beiden Gruppen. Die routinemäßige prophylaktische Einnahme von Vitamin E empfehlen wir daher aktuell nicht, bis entsprechend Daten aus weiteren doppelblind randomisierten klinischen Studien, die die präventive Wirkung von Vitamin E untersuchen, vorliegen [10].

In Tab. 1 ist am Beispiel der immuntherapieassoziierten Neuropathie das stufenweise diagnostische und therapeutische Vorgehen dargestellt.

**Table 1:** 

Toxizitätsgrad nach CTC-AE-Kriterien	Diagnostik und Verlaufskontrolle	Behandlung
*Grad I: *asymptomatischer Befund, milde Symptome, keine Funktionseinschränkung	Klinisch-neurologische Untersuchung, PNP-Labor (Vitamin B12, Folsäure, Diabetes und HIV-Screening), cMRT, sMRT oder Plexusdarstellung mit und ohne Kontrastmittelgabe, elektrophysiologische Diagnostik	Fortführung der Immuntherapie möglich, ggf. Dosisreduktion oder Pause
*Grad II: *mittelgradige Symptome, leichte Beeinträchtigung im Alltag, Hirnnervenbeteiligung	Wie bei Grad I zusätzlich Lumbalpunktion, Lungenfunktionsprüfung	Immuntherapie pausieren
		Methylprednisolon 0,5–1 mg/kg Körpergewicht/Tag i.v. über 5 Tage
	4‑wöchige neurologisch/neuroonkologische Verlaufskontrolle bis zum vollständigen Abklingen bzw. möglichst Besserung Grad I	→ Nach Therapieansprechen: dosisäquivalente orale Prednisolongabe und schrittweise Ausschleichen über 4–6 Wochen
	CAVE: bei Rebound-Phänomen unter Reduktion bzw. Verschlechterung nach Therapieansprechen, ggf. Eskalation erwägen	→ Bei fehlendem Therapieansprechen: wie Grad III–IV behandeln
*Grad III–IV: *schwere Symptome, potenziell lebensgefährliche Nebenwirkungen, z. B. Dyspnoe	Wie bei Grad I und II	Immuntherapie absetzen, stationäre Aufnahme (Je nach Manifestation und Dringlichkeit, strenge Nutzen-Risiko-Abwägung bei der Reevaluation – nur im Falle vollständiger Erholung neurologischer Erkrankung)
	Neuro(onko)logische stationäre Behandlung, 4‑wöchige Verlaufskontrolle in neuroonkologischer Ambulanz bis zum vollständigen Abklingen bzw. möglichst Besserung Grad I	
	CAVE: bei Rebound-Phänomen unter Reduktion bzw. Verschlechterung nach Therapieansprechen ggf. Eskalation erwägen	Methylprednisolon 2 mg/kg Körpergewicht / Tag i.v. über 5 Tage
		→ Nach Therapieansprechen: 1 mg/kg Körpergewicht /Tag orale Prednisolongabe und schrittweise Ausschleichen über 4–6 Wochen
		→ Bei fehlendem Ansprechen bzw. Rebound-Phänomen unter Steroidreduktion: individuelles Erwägen weiterer Immunmodulation (z. B. IVIG, Methotrexat, Infliximab, Tocilizumab) je nach neurologischer Manifestation, Organbeteiligung und onkologischem Staging
		→ Bei fehlendem Ansprechen Anti-CD-20-Antikörper erwägen [13, 14]

*cMRT* kranielle Magnetresonanztomographie, *CTC AE* Common Terminology Criteria for Adverse Events, *HIV* humanes Immundefizienzvirus,* IVIG* intravenöse Immunglobuline, *PNP* Polyneuropathien, *sMRT* spinale Magnetresonanztomographie

### Myopathien, Myositiden, myasthene Syndrome

Patientinnen und Patienten mit Krebserkrankungen schildern häufig subjektive Beschwerden im Bereich der Muskulatur. Diese können auf **tumorbedingte Kachexie**

tumorbedingte Kachexie

und/oder auf **längerfristige Steroideinnahme**

längerfristige Steroideinnahme

zurückführbar sein, daher gilt es stets, bei diesem Beschwerdekomplex eine sorgfältige **Ernährungsberatung**

Ernährungsberatung

zu veranlassen und die Steroiddosis zu reevaluieren, zudem nach Fatigue und körperlicher Aktivität zu fragen. Die therapieassoziierten Myopathien, die unter Immuncheckpoint-Inhibitoren (z. B. PD-1-Antikörper oder CTLA4-Antikörper) oder unter dem Proteinkinaseinhibitor Imatinib oder dem MEK1/2(„mitogen activated protein kinase 1 and 2“)-Inhibitor Selumetinib auftreten, können allerdings auch die **Rumpfmuskulatur**

Rumpfmuskulatur

betreffen [10], daher ist eine sorgfältige klinische Evaluation von entscheidender Bedeutung für eine zielführende (Differenzial‑)Diagnose.

Das diagnostische Vorgehen entspricht im Wesentlichen den Leitlinien der Deutschen Gesellschaft für Neurologie. Eine **Muskelbiopsie**

Muskelbiopsie

kann bei unklarer Faktenlage helfen, tumorbedingte vs. therapieassoziierte vs. andere Myopathien zu differenzieren. Es ist zudem wichtig, eine **kardiologische Abklärung**

kardiologische Abklärung

nicht zu vergessen, da gerade Immuntherapien zu Myokarditiden und Kardiomysositiden führen können [15, 16].

Therapieassoziierte myasthene Syndrome, die auch bereits durchaus frühzeitig als therapieassoziierte neurologische Symptome, insbesondere unter Immuntherapien auftreten können, ähneln den „regulären“ Erscheinungsformen dieser Erkrankungen. Entsprechend sind diagnostisches und therapeutisches Vorgehen ähnlich und gemäß den Leitlinien der Deutschen Gesellschaft für Neurologie vorzunehmen (siehe auch Tab. 2).

**Table 2:** 

Syndrom (Verdachtsdiagnose)	Diagnostik und Verlaufskontrolle	Behandlung
*Guillain-Barré-Syndrom*	Umgehende Diagnostik und rasche Therapieeinleitung!	Beim Verdacht auf therapieassoziiertes GBS: Methylprednisolon 1–2 mg/kg Körpergewicht Frühzeitig IVIG erwägen Intensivüberwachung
	Elektrophysiologische Diagnostik Lumbalpunktion (erhöhtes Protein, normale Zellzahl, Antikörpertestung für GBS-Varianten) Lungenfunktion mit Vitalkapazität	
*Myasthenia-gravis/myasthene-Syndrome*	Neurologische Untersuchung, besonderer Fokus auf Okulomotorik und proximale Muskelgruppen	Methylprednisolon 1–2 mg/kg Körpergewicht i.v. über 5 Tage
	Tensilontest	Pyridostigmin, 30 mg initiale Dosis
	Elektrophysiologische Diagnostik (repetitive Stimulation)	Bei Nichtbesserung: Plasmapherese oder IVIG-Gabe
		Weitere Immunsuppression z. B. mit Azathioprin oder Cyclophosphamid
*Aseptische Meningitis und/oder Enzephalitis*	Ausschluss Hirndruck, cMRT und sMRT mit und ohne Kontrastmittelgabe *vor *Lumbalpunktion (Eröffnungsdruck, Zellzahl und Basisdiagnostik inkl. IgG-Index, Zytologie/Neuropathologie, PCR für HSV, VZV, Mikrobiologie: Borrelien) Blutkulturen	Methylprednisolon 2 mg/kg Körpergewicht oder bei schwer betroffenem PatientInnen 500–1000 mg über 5 Tage nach Erregerdiagnostik, bei noch ausstehenden virologischen und mikrobiologischen Befunden zusätzlich i.v. Aciclovir und Antibiose nach DGN-Leitlinie
	EEG	
	Beim Verdacht auf Enzephalitis metabolische Ursachen evaluieren (z. B. Kalzium, Ammoniak, TSH)	
	Seltene Antikörper (NMDA-Rezeptor-Antikörper oder paraneoplastische, onkoneurale Antikörper wie z. B. Anti-Hu, Anti-CASPR2, Anti-NMDA-Rezeptor) berücksichtigen [2, 17, 18]	
*Transverse Myelitis*	cMRT und sMRT mit und ohne Kontrastmittelgabe vor Lumbalpunktion	Methylprednisolon 1–2 mg/kg Körpergewicht Frühzeitig IVIG und Plasmapherese erwägen
	Serum-Vitamin B12, HIV, Syphilis, ANA, Anti-Ro- und, Anti-La-Antikörper, Anti-Aquaporin4-IgG, TSH	
	Elektrophysiologische Diagnostik	
*Myopathie*	Labor: CK, CK-MB, Troponin, Transaminasen	Methylprednisolon 1–2 mg/kg Körpergewicht
	Elektrophysiologie (EMG, NLG, repetitive Stimulation)	
	EKG, Herzechokardiogramm	
	Muskelbiopsie	

*CK* Kreatinkinase, *cMRT* kranielle Magnetresonanztomographie, *DGN* Deutsche Gesellschaft für Neurologie,* EKG* Elektrokardiographie, *EMG* Elektromyographie, *GBS* Guillain-Barré-Syndrom, *HIV* humanes Immundefizienzvirus,* IVIG* intravenöse Immunglobuline,* HSV* Herpes-simplex-Virus, *IgG* immunglobulin G, *NLG* Nervenleitungsgeschwindigkeit, *NMDA* N-Methyl-D-Aspartat,* PCR* „polymerase chain reaction“, *sMRT* spinale Magnetresonanztomographie, *TSH* thyreoideastimulierendes Hormon, *VZV* Varizella-Zoster-Virus

## Zentrales Nervensystem

### Hypophysitis

Die Hypophysitis gehört zu den prominenten und häufigsten therapieassoziierten neurologischen Symptomen unter **Immuncheckpoint-Inhibition**

Immuncheckpoint-Inhibition,

hier insbesondere unter dem CTLA4-Inhibitor **Ipilimumab**

Ipilimumab,

deutlich seltener unter PD-1-/PD-L1-Blokade. Die Hypophysitis tritt bei Ipilimumabbehandlung bei knapp 10 % der behandelten Patientinnen und Patienten bereits innerhalb der ersten 6 bis 8 Wochen auf [19]. Die Diagnose kann dadurch erschwert werden, dass die Beschwerden der PatientInnen sehr unspezifisch sein können. Wir empfehlen daher, die **Differenzialdiagnose**

Differenzialdiagnose

einer Hypophysitis bei stattgehabter Immuncheckpoint-Inhibition zu konsiderieren und sorgfältig abzuklären, dies zudem auch bei unklaren therapieassoziierten neurologischen Symptomen unter anderen Tumortherapien.

### Enzephalopathien

Enzephalopathien können durchaus rasch nach dem Therapiestart mit klassischen zytotoxischen Zytostatika auftreten und somit in der Regel unschwer als therapieassoziiert erkannt werden, z. B. induziert durch das Alkylanz **Ifosfamid**

Ifosfamid

bzw. durch intrathekale Applikation des Folsäureantagonisten **Methotrexat**

Methotrexat.

Therapieassoziierte Enzephalopathien im Zusammenhang mit medikamentösen Systemtherapien oder auch intrathekalen Therapien, die nach einer **Ganzhirnbestrahlung**

Ganzhirnbestrahlung

auftreten, haben häufig schwere und irreversible Verläufe [20].

Auch strahlentherapieinduzierte Effekte unter Checkpoint-Inhibition sollten in Betracht gezogen werden: Hierbei kann es vermehrt zum sog. **abskopalen Effekt**

abskopalen Effekt

kommen. Dies bedeutet, dass z. B. nach einer extrakraniellen Bestrahlung ein intrakranielles Ansprechen durch eine radiotherapieinduzierte Immunmodulation beobachtet werden kann [21].

Auch die modernen zielgerichteten onkologischen Therapien, z. B. **Proteasominhibitoren**

Proteasominhibitoren

(z. B. Bortezomib), können akute Enzephalopathien verursachen [22], ebenso Immuncheckpoint-Inhibitoren und **CAR‑T-Zellen**

CAR-T-Zellen.

Der Entstehungsmechanismus dieser verschiedenen Formen therapieassoziierter Enzephalopathien ist jeweils verschieden. Wichtig und allen Formen gemeinsam ist, dass eine Abgrenzung zu **erregerbedingten**

erregerbedingten

(vor allem viralen) Enzephalitiden rasch erfolgen muss. Wir empfehlen bei einer entzündlichen Liquorkonstellation bis zum negativen HSV(Herpes-simplex-Virus)- und VZV(Varizella-Zoster-Virus)-PCR(„polymerase chain reaction“)-Test eine **Aciclovirtherapie**

Aciclovirtherapie

zu initiieren (siehe auch Tab. 2).

### Aseptische Meningitis

Zwar ist die Diagnose einer aseptischen Meningitis dadurch erschwert, dass es keine beweisenden diagnostischen Befunde gibt. Umso wichtiger ist es, diese bei unklarer Liquorpleozytose und fehlendem Erregernachweis stets differenzialdiagnostisch zu erwägen. **Erhöhte Zytokinwerte**

Erhöhte Zytokinwerte

im Liquor können hilfreich sein, ebenso eine bildmorphologische **Kontrastmittelanhebung der Meningen**

Kontrastmittelanhebung der Meningen.

Dokumentierte Beobachtungen aseptischer Meningitiden gibt es unter Immuncheckpoint-Therapie [23] und auch unter dem Multikinaseinhibitor Regorafenib [24]. In der Regel ist dieses Krankheitsbild **steroidresponsiv**

steroidresponsiv,

sodass eine Prednisolontherapie bereits zügig zu einer Verbesserung führen kann (siehe auch Tab. 2).

## Therapiestrategien

Die nationalen und europäischen **Fachgesellschaften**

Fachgesellschaften

aktualisieren fortlaufend die Empfehlungen für die **Behandlungsstrategien**

Behandlungsstrategien

therapieassoziierter neurologischer Symptome. Dies trifft sicherlich am weitesten für die Immuncheckpoint-Inhibitoren und für die CAR-T-Zellen zu. Wir empfehlen daher die alerte und **zügige neurologische Evaluation**

zügige neurologische Evaluation

und Differenzialdiagnostik und die Zusammenarbeit mit einem **zertifizierten neuroonkologischen Zentrum**

zertifizierten neuroonkologischen Zentrum.

Zu betonen ist, dass einige der therapieassoziierten neurologischen Symptome, insbesondere unter den Immuntherapien, sehr rasch lebensbedrohlich werden können. Da sie aber meistens gleichzeitig auch therapierbar sind, obliegt den erstevaluierenden ärztlichen Kolleginnen und Kollegen eine sehr hohe Verantwortung, die Situation richtig einzuordnen und die entsprechenden Schritte zügig einzuleiten. Konzeptionell dargelegt bestehen die wichtigsten Maßnahmen aus dem **Absetzen der auslösenden Medikation**

Absetzen der auslösenden Medikation,

einer **engmaschigen klinischen Evaluation**

engmaschigen klinischen Evaluation,

bei Persistenz oder Verschlechterung müssen dann je nach Pathomechanismus eine Prednisolontherapie, intravenöse Immunglobuline, Azathioprin, Tocilizumab oder auch eine Plasmapherese konsideriert werden.

Die Therapiedauer ist abhängig von der klinischen Symptomatik und Entwicklung der jeweils im Vordergrund stehenden laborchemischen, liquordiagnostischen und/oder bildgebenden Diagnostik (siehe Tab. 1 und 2).

## Fazit für die Praxis

- In der Behandlung von Tumorerkrankungen ist die Verlängerung von progressionsfreiem und Gesamtüberleben ein vorrangiges Ziel. Gleichermaßen wichtig ist die berichtete Lebensqualität, denn therapieassoziierte neurologische Symptome können nicht nur die Therapiedurchführung, sondern insbesondere die Lebensqualität der PatientInnen stark beeinträchtigen.
- Es wird künftig wichtig sein, Biomarker zu identifizieren, die helfen, das Auftreten therapieassoziierter (neurologischer) Symptome vorherzusehen und eine entsprechende Stratifizierung bei der Therapieplanung vorzunehmen.
- Zudem formulierte aktuell eine Expertengruppe für bestimmte Tumorentitäten und ausgewählte Therapieformen die Einführung eines neuen klinischen Endpunkts, das „severe toxicity-free survival“, d. h. der zeitliche Abstand zwischen Therapiestart und Auftreten einer schweren therapieassoziierten Symptomatik. Dieser Endpunkt gibt eine wichtige Richtung für die Weiterentwicklung onkologischer Therapien vor.

## CME-Fragebogen

Eine tumortherapieassoziierte Polyneuropathie …

wird nicht durch Checkpoint-Inhibitoren verursacht.

tritt nur unter Vincristintherapie auf.

sistiert unmittelbar nach Abbruch der Therapie.

kann durch frühzeitige Vitamin-E-Gabe verhindert werden.

kann bei vorbestehender diabetischer Neuropathie verstärkt werden.

Bei systematischer klinischer und diagnostischer Vorgehensweise bei neurologischen Beschwerden von TumorpatientInnen ist folgende Maßnahme *nicht* ausreichend:

Neurologische Untersuchung

Laborchemische Untersuchung inklusive Zytokine

Natives kranielles Computertomogramm

Liquordiagnostik

Medikamentenanamnese

Welche Fachdisziplin sollte bei PatientInnen mit Tumorerkrankungen mit Beschwerden im Bereich der Muskulatur konsultiert werden?

Orthopädie wegen Myegelosen

Gastroenterologie wegen Gastroparese

Hämatologie wegen Osteomyelitis

Kardiologie wegen möglicher Myokarditis

Urologie wegen Blasenmuskelstörung

Welche der folgenden Tumortherapien führt am häufigsten zu neurologischen Symptomen im peripheren Nervensystem?

CTLA4 („cytotoxic T lymphocyte-associated antigen 4“)-PD1 („programmed cell death protein 1“)/PDL1 („programmed death-ligand 1“)-Antikörper (Ipilimumab, Nivolumab, Pembrolizumab, Atezolizumab)

CDK („cyclin-dependent kinase“)-4/6-Inhibitoren (Palbociclib, Abemaciclib, Ribociclib)

EGFR („epidermal growth factor receptor“)-Inhibitoren (Afatinib, Neratinib)

VEGF („vascular endothelial growth factor“)-Antikörper (Bevacizumab)

FGFR („fibroblast growth factor receptor“)-Inhibitoren (Erdafitinib, Pemigatinib)

Wie gehen Sie beim Verdacht auf eine Tumorprogression in das Zentralnervensystem als Ursache einer neurologischen Symptomatik einer Tumorpatientin/eines Tumorpatienten vor?

Primär Immunglobuline i.v.

Deeskalation der Therapie, da infauste Situation

Interdisziplinäre Diskussion im Tumorboard

Ganzhirnbestrahlung planen

Umgehend hochdosiert Steroide geben

Ein 65-jähriger Patient stellt sich montags in Ihrer Praxis mit neuen, über das Wochenende aufgetretenen Paresen der unteren Extremitäten Er berichtet von allgemeinem Unwohlsein, Müdigkeit und Abgeschlagenheit. Vor allem beim Aufstehen aus sitzender Position und beim Treppensteigen verspüre er eine deutliche Schwäche der Beine. In der Medikamentenanamnese erfahren Sie, dass er wegen eines malignen Melanoms mit dem Anti-PD1(„programmed cell death protein 1“)-Antikörper Nivolumab behandelt wird, er habe zudem kurzzeitig ein Antibiotikum eingenommen, dessen Name er nicht erinnert, es sei wegen eines Harnwegsinfekts gewesen. In der klinisch-neurologischen Untersuchung fallen Ihnen folgende Befunde auf: Hüftabduktion beidseits Kraftgrad 4, Achillessehnen- und Patellarsehnenreflex beidseits erloschen. Wie gehen Sie vor?

Stationäre Aufnahme in die Klinik zur elektrophysiologischen Diagnostik, spinale Magnetresonanztomographie und Lumbalpunktion

Beim Verdacht auf immuntherapieassoziierte Nebenwirkungen milder Ausprägung Beginn mit 0,5 mg/kg Körpergewicht Prednisolon und Wiedervorstellung in 3 Tagen

Vorstellung beim Onkologen des Patienten bei nächster Routinekontrolle

Urinstatus und Thoraxröntgen veranlassen

Ergänzung einer elektrophysiologischen Diagnostik in Ihrer Praxis am Folgetag

Eine 48-jährige Patientin mit malignem Melanom unter kombinierter Immuntherapie mit den Checkpoint-Inhibitoren Nivolumab und Ipilimumab präsentiert sich mit Kopfschmerzen, Desorientiertheit und zunehmender Fatigue. Welche Reihenfolge der Diagnostik und Therapie ist sinnvoll?

Kranielle Magnetresonanztomographie (cMRT) und spinale Magnetresonanztomographie (sMRT), Lumbalpunktion, Beginn Aciclovir und Steroide, Elektroenzephalographie (EEG) und weitere Ausbreitungsdiagnostik

Direkt mit hochdosierter Steroidtherapie beginnen

Ausbreitungsdiagnostik für die Tumorerkrankung durchführen, danach cMRT/sMRT

Klinische Verlaufskontrolle in 1 Woche, bis dahin Checkpoint-Inhibition pausieren

Lumbalpunktion und Beginn mit 3‑fach antibiotischer Therapie beim Verdacht auf Meningitis nach Leitlinie

Die kranielle Magnetresonanztomographie (cMRT) bei einem Patienten mit zerebral metastasiertem „non-small-cell lung cancer“ (NSCLC) zeigt nach Kontrastmittel(KM)-Gabe eine Signalanhebung im Bereich der Nn. faciales. Klinisch-neurologisch besteht eine progrediente Hypakusis, Fatigue, aktuelle tumorspezifische Therapie seit 4 Wochen eine Kombination u. a. mit Pembrolizumab. Zudem hat der Patient eine Ganzhirnbestrahlung vor 8 Wochen erhalten. Bei einer Lumbalpunktion ergibt sich eine mäßige Liquorpleozytose. Welche Differenzialdiagnose ist am wahrscheinlichsten?

Bakterielle Meningitis

Toxoplasmose

Aseptische Meningitis

Polyneuritis cranialis

Reizpleozytose nach Ganzhirnbestrahlung

Welche Aussage zur Therapie mit Checkpoint-Inhibitoren ist *nicht* richtig?

Checkpoint-Inhibitoren können bereits nach wenigen Wochen zu therapieassoziierten neurologischen Symptomen führen.

Bei der Verdachtsdiagnose eines therapieassoziierten neurologischen Symptoms unter Checkpoint-Inhibition erfolgt umgehend eine Steroidtherapie.

Checkpoint-Inhibitoren können zu Hypophysitis führen, dies wurde häufiger unter CTLA4(„cytotoxic T lymphocyte-associated antigen 4“)-Blockade (als unter PD1[„programmed cell death protein 1“]-Blockade) beobachtet.

Checkpoint-Inhibitoren sind bei einigen Tumorentitäten sehr effektiv und führen zu Langzeitüberleben.

Checkpoint-Inhibitoren können auch zur Behandlung von ZNS (Zentralnervensystem)-Metastasen eigesetzt werden.

Was trifft für eine Kombination aus Ganzhirnbestrahlung mit medikamentöser Tumortherapie zu?

Kann zur schweren und irreversiblen Enzephalopathie führen

Darf wegen möglicher Enzephalopathie nicht eingesetzt werden

Ist bei primären ZNS (Zentralnervensystem)-Malignomen Standardtherapie

Ist ohne Gefahr neurokognitiver Leistungseinbußen anwendbar

Kommt nur als Ultima Ratio zum Einsatz
